# Efficacy and safety of lopinavir/ritonavir (LPV/r) in antiretroviral-experienced subjects infected with different subtypes of HIV-1

**DOI:** 10.1186/1758-2652-13-S4-P30

**Published:** 2010-11-08

**Authors:** L Maroldo, LM Fredrick, K Robinson-Morgan, R Trinh, TJ Podsadecki

**Affiliations:** 1 Abbott, Abbott Park, USA

## Purpose

There are three classes of HIV-1 based on diversity of the viral envelope: M (major), O (outlying) and N (new). The M group is subclassified into nine major subtypes including A—D, F—H, J and K, as well as several recombinant forms. There is a growing need to evaluate antiretroviral (ARV) treatment in these diverse subtypes.

## Methods

M06-802 was an open-label, global, 48-week phase III trial. 599 ARV-experienced, HIV-1-infected subjects were randomized 1:1 to receive LPV/r 800/200mg QD or 400/100mg BID with ≥2 investigator-selected nucleoside/nucleotide reverse transcriptase inhibitors. Classification of HIV-1 viruses by subtype was determined by analyzing sequences of the *env* gene for this post hoc analysis.

## Results

**Figure 1:**
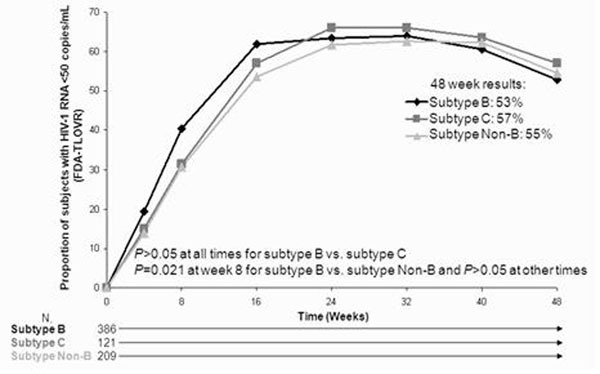
Percent of subjects infected with different subtypes that have plasma HIV-1 RNA <50 copies/mL, FDA-TLOVR analysis

Of the 595 subjects with available sequence data, 386 (65%) were infected with subtype B, 121 (20%) were infected with subtype C, 88 (15%) were infected with another subtype. Subjects infected with subtype B were more likely to be male (75%), white (56%), and living in North America (N.A.) (52%), while subjects infected with subtype C were more likely to be female (56%), black (83%), and living outside of N.A. or Western Europe (W.E.) (99%). Subjects infected with other subtypes were more likely to be male (56%), white (88%) and living outside of N.A. or W.E. (82%). The proportion of subjects with subtype B responding to treatment at week 48 as analyzed using the FDA-TLOVR method (53%) was similar to that of subtype C subjects (57%, *P*=0.465) and non-B subtype subjects (55%, *P*=0.731) (Figure 1). The prevalence of moderate/severe treatment-related adverse events was similar across subtypes. There was a nonsignificant trend to more frequent emergence of new protease resistance mutations in subtype B (18%) vs. subtype C (8%, *P*=0.277) or non-B (7%, *P*=0.088) subjects; however, subtype B subjects were more likely to have previously been treated with PIs.

## Conclusion

LPV/r demonstrated similar efficacy and safety among subjects infected with different HIV-1 subtypes.

